# Antiplasmodial activity of *Vernonia adoensis* aqueous, methanol and chloroform leaf extracts against chloroquine sensitive strain of *Plasmodium berghei* in vivo in mice

**DOI:** 10.1186/s13104-018-3835-2

**Published:** 2018-10-17

**Authors:** Gebreyohannes Zemicheal, Yalemtsehay Mekonnen

**Affiliations:** Department of Biology, College of Natural and Computational Sciences, Addis Ababa University, P.O.Box 1176, Addis Ababa, Ethiopia

**Keywords:** *Vernonia adoensis*, *Plasmodium berghei*, Antiplasmodial, Parasitaemia, Chloroquine

## Abstract

**Objective:**

The aim of this study was to investigate the antiplasmodial effects of the crude aqueous, methanol and chloroform extracts of the leaves of *Vernonia adoensis* in *Plasmodium berghei* infected Swiss albino mice using Peters’ 4-day suppressive test.

**Results:**

The number of mice used for the toxicity test was 20 (5/group) and for each extract and control groups 5 mice per group was used. The aqueous, methanol and chloroform extracts of *V. adoensis* leaves indicated statistically significant (P < 0.05) suppression of parasitaemia in the treated mice. The highest inhibition was that of the methanol extract treated mice (83.36%) followed by aqueous (72.26%) and chloroform (54.34%) at an oral dose of 600 mg/kg b.wt. Each extract prevented body weight loss and packed cell volume (PCV) reduction as compared to the negative control groups. The survival time of the mice treated with chloroform based on Kaplan–Meir analysis was 12.53 ± 0.37 at 600 mg/kg b.wt, while the negative control was 7.93 ± 0.37 days. The LD_50_ of the extracts was greater than 3000 mg/kg body weight. In conclusion, the crude leaves extract of *V. adoensis* have demonstrated antiplasmodial effect in vivo. *P. berghei* infection is suppressed in a dose-dependent manner showing relevance of the traditional use of the plant.

**Electronic supplementary material:**

The online version of this article (10.1186/s13104-018-3835-2) contains supplementary material, which is available to authorized users.

## Introduction

Malaria is still a public health problem in many parts of the tropics. *Plasomodium falciparum* and *Plasomodium vivax* are the most fatal species [1–5]. Currently quinine and artemisinin are the two effective drugs obtained from two traditional medicinal plants. Quinine was obtained from the bark of the *Cinchona* tree [6, 7] and artemisinin from the plant *Artemisia annua* [8, 9]. However, in the recent years these drugs show some degree of resistance [10, 11].

*Vernonia adoensis* (*V. adoensis*) is among the numerous traditionally used medicinal plants in Ethiopia. It is used for malaria, gastro-intestinal complaints, muscle spasm and for healing wounds [12–15]. In Tanzania a root-infusion is taken for stomach-pain and as anti-tuberculosis and fresh roots sliced and cooked is taken with milk against gonorrhoea [12]. Previously the antimicrobial [9], antioxidant and antipyretic [14] property of *V. adoensis* is reported.

In this study the antiplasmodial effect of the leaf crude extract of *V. adoensis* against chloroquine sensitive *P. berghei* in Swiss albino mice is tested.

## Main text

### Plant material collection

Fresh leaves of *V. adoensis* were collected from Gondar town that grows freely around the Gondar Hospital, Ethiopia during the month of October, 2012. Field study and any plant material collection took place upon official authorization in accordance with country’s laws and following international guidelines. The identification and authentication of the plant specimen was carried out by Professors Ensermu Kelbessa and Sebsebe Demissew at the National Herbarium of the Addis Ababa University (AAU). A voucher specimen number GZ 02/2012 of the plant sample was placed at the Herbarium.

### Preparation of crude plant extracts

The preparation of crude extracts was done based on our previous published works [16, 17]. In summary the collected plant leaves of *V. adoensis* were cleaned and air dried at room temperature under a shade in the Biomedical Science Laboratory of AAU. An electrical grinding mill (IEC, 158 VDE 0660, Germany) was used to grind the leaves. Sensitive digital weighing balance (AND: FX-320, Japan) was used to weigh the powdered plant material. Each extract was prepared in 1:10 ratio (w/v). Measurement of the percentage yield of each extract was done.

#### Experimental animals

We obtained the Swiss albino mice (25–34 g) from the Animal House of the College of Natural Sciences of AAU. Standard pellet diet and tap water ad libitum are fed to the mice housed in standard cages. All experiments were done three times and the tables represent the mean of the three experiments in each case. The Ethics Committee of the College of Natural Sciences of Addis Ababa University gave approval to run the experiment.

### Toxicity test

Acute toxicity test (single dose exposure) of aqueous, methanol and chloroform extracts from the leaves of *V. adoensis* were evaluated in 3-h fasted Swiss albino mice through an oral administration of 2000, 2500 and 3000 mg/kg body weight of mice. The Organization for Economic Cooperation and Development (OECD) guidelines 425 procedure was followed to test the toxicity.

### Evaluation of the antiplasmodial activity

In the in vivo evaluation of the antiplasmodial activity of the plant extracts was employed against chloroquine (CQ) sensitive *P. berghei* in mice using the standard 4-day suppressive method [18]. Gentle heart puncture from donor mouse and anesthetized with chloroform afforded 1 ml blood the rising parasitaemia being about 33%. Then, the 1 ml blood was diluted with 4 ml of physiological saline and thus, one ml blood contains about 5 × 10^7^ infected red blood cells. The procedure we followed is previously given in Tekalegn et al. [16] and Zerihun et al. [17]. Twenty-five male mice were infected with *P. berghei* and randomly divided into five groups of five mice per group. Three test groups and two control groups (dH_2_O or 3% Tween 80 as a negative control CQ as positive control). Each mouse was inoculated on day 0, (D_0_) (intraperitoneally) with 0.2 ml of infected blood having approximately 1 × 10^7^ *P. berghei* parasitized red blood cells as standard inoculum. The different doses given were 200 mg/kg, 400 mg/kg and 600 mg/kg of body weight and 0.2 ml CQ at 25 mg/kg. Standard intragastric tube was used to give the extracts and the controls. Treatment was started after 3 h of infection on D_0_ and then following four consecutive days in 24 h schedule.

On the 5th day Blood sample was collected from tail snip of each mouse on the 5th day. Thin smears preparation is as given previously [16, 17]. The percentage parasitaemia and suppression was calculated as:

$${\text{Percentage parasitaemia }} = \frac{\text{Number of parasitized red blood cells}}{\text{Total number of RBC examined}} \, ({\text{RBC}}) \, \times { 1}00$$

$${\text{Percentage suppression }} = \frac{\text{Parasitaemia in negative control}}{\text{Parasitaemia in negative control}} \, - {\text{ Parasitaemia in treated}} \times 100$$

### Determination of body weight and packed cell volume (PCV)

The body weight and PCV of each mouse in all the groups was recorded before infection and after infection. The average body weights were calculated as:

$${\text{Mean body weight }} = \frac{\text{Total weight of mice in a group}}{\text{Total number of mice in that group}}$$

Heparinized microhematocrit capillary tubes up to 3/4th of their length was used to collect blood from tail of each mouse for PCV measurement. The tubes were sealed by crystal seal and placed in the microhematocrit centrifuge (Microheamatocrit Centrifuge, 583298, Hawksley & Sons Ltd, England) with the sealed ends out wards. The sample was centrifuged at 12,000 rpm for 4 min. The volume of the total blood and the volume of red blood cells were measured and PCV was calculated as:

$${\text{PCV }} = \frac{\text{Volume of erythrocytes in a given volume of blood}}{\text{Total blood volume}} \times 100$$

### Determination of mean survival time

Mortality was observed daily and the number of days from the time of inoculation of the parasite up to death was recorded for each mouse in the treatment and control groups throughout the follow up period. The mean survival time (MST) for each group was calculated using the SPSS version 20 applying Kaplan–Meir statistical analysis.

### Data analysis

Results of the study were expressed as mean ± standard error of the mean (M ± SEM). Data obtained from the parasitaemia, body weight, PCV and survival times were analyzed using Windows SPSS Version 15. The one-way analysis of variance (ANOVA) and paired-samples Student’s *t* test were used to compare results among and within groups for differences between initial and final results. The result was considered statistically significant at 95% confidence level and P-value < 0.05.

### Results

#### Toxicity test

No death occurred in any of the groups and dose levels during the entire period of 24 h of observation. Furthermore, during the gross physical and behavioral observation of the experimental mice, for mice treated groups with the aqueous and methanol (99.5%) extracts at the dose of 3000 mg/kg showed some reduction in feeding activity, hair erection and rigidity after the administration of the doses. In general, the oral administrations of the aqueous, methanol and chloroform extracts of *V. adoensis* in each doses of 2000, 2500 and 3000 mg/kg did not produce any significant changes in usual behavior and also no mortality. Therefore, the LD50 is greater than 3000 mg/kg body weight.

#### Antiplasmodial activity

The results of this study indicated that the in vivo aqueous, methanol and chloroform leaf extracts of *V. adoensis* exhibited a potent activity against *P. berghei* malaria parasite (Table 1). The Parasitaemia suppressive effects produced by all the test extracts were significant (P < 0.05) as compared with their respective negative control groups. The highest percent suppression at 600 mg/kg body weight was 83.36%. The mice treated with the standard drug (chloroquine 25 mg/kg) were completely free from the parasites in all the experiments using the aqueous, methanol and chloroform leaf extracts of *V. adoensis* (Table 1).

**Table 1:** Antiplasmodium activity of aqueous, methanol and chloroform extract of *V. adoensis* leaves against *P. berghei* in Swiss albino mice

Test extracts	Dose (mg/kg/day)	Antiplasmodial activity	
		% Parasitaemia ± SEM	% Suppression ± SEM
Aqueous	NC	25.79 ± 1.00^a^	0.00^a^
	200	15.72 ± 0.77^b^	39.04 ± 2.99^b^
	400	12.21 ± 0.81^b^	52.62 ± 3.16^c^
	600	7.15 ± 0.94^c^	72.26 ± 3.67^d^
Methanol	NC	21.23 ± 0.49^a^	0.00^a^
	200	10.58 ± 0.83^b^	50.17 ± 3.91^b^
	400	5.79 ± 0.96^c^	72.71 ± 4.52^c^
	600	3.53 ± 0.23^c^	83.36 ± 1.12^c^
Chloroform	NC	27.66 ± 0.89^a^	0.00^a^
	200	19.06 ± 0.46^b^	31.09 ± 1.66^b^
	400	16.41 ± 0.46^c^	40.65 ± 1.60^c^
	600	12.62 ± 0.76^d^	54.34 ± 2.78^d^
	PC	0.00^e^	100.00^e^

Values are presented as Mean ± SEM

PC, positive control; NC, negative control (0.2 ml of 3% Tween 80)

^a,b,c,d,e^Values in the same column followed by the same letter do not differ significantly (P > 0.05)

### Body weight and packed cell volume (PCV)

The plant extracts prevented body weight loss and the mean body weight on D_0_ and day 4 (D_4_) did not show statistically significant difference (P > 0.05) except at lower dose level of 200 mg/kg in both aqueous and chloroform extracts. Therefore, the prevention of body weight reduction by each extracts was dose dependent. Body weight of the mice in the extract treated groups on D_4_ is significantly higher (P < 0.05) than that of the mice in the negative control group (Table 2).

**Table 2:** Effect of *V. adoensis* of aqueous, methanol and chloroform leaf extracts on the body weights of *P. berghei* infected mice

Test extracts	Dose (mg/kg/day)	Body weight		% Change
		Before treatment (D_0_)	After treatment (D_4_)	
Aqueous	NC	28.22 ± 0.42	24.66 ± 0.29*	− 12.62^a^
	200	27.74 ± 0.21	25.66 ± 0.52*	− 7.49^b^
	400	29.08 ± 0.28	28.88 ± 0.71	− 0.68^b^
	600	29.62 ± 0.18	28.46 ± 0.49	− 3.92^b^
	PC	29.66 ± 0.38	28.82 ± 0.16	− 2.83^b^
Methanol	NC	28.92 ± 0.30	25.82 ± 0.09*	− 10.72^a^
	200	30.60 ± 0.31	28.53 ± 0.83	− 6.76^b^
	400	30.08 ± 0.34	29.2 ± 0.68	− 2.93^b^
	600	28.5 ± 0.36	28.2 ± 0.40	− 1.05^b^
	PC	27.27 ± 0.26	27.12 ± 0.24	− 0.55^b^
Chloroform	NC	26.58 ± 0.47	22.72 ± 0.27*	− 14.52^a^
	200	51.67 ± 0.25	51.02 ± 0.46	− 1.26^b^
	400	52.3 ± 0.36	52.12 ± 0.33	− 0.34^b^
	600	53.16 ± 0.63	53.13 ± 0.64	− 0.06^b^
	PC	51.42 ± 0.35	52.08 ± 0.55	1.28^b^

Values are presented as Mean ± SEM

NC, negative control (0.2 ml of respective vehicle); PC, positive control (0.2 ml of CQ)

* There was significant change between D_0_ and D_4_ (P < 0.05)

^a,b^Values in the same column followed by the same letter do not differ significantly (P > 0.05)

Table 3 shows that the test extract doses of *V. adoensis* prevented PCV reduction due to parasitaemia infection. Each result showed that PCV in the respective negative control groups were significantly (P < 0.05) reduced in D_4_ while in the extracts treated groups significant change was not observed (P > 0.05). Furthermore, the analysis of variance performed between the extract treated groups in comparison with the corresponding negative control groups showed highly significant variation.

**Table 3:** Effect of *V. adoensis* of aqueous, methanol and chloroform leaf extracts on the packed cell volume of *P. berghei* infected Swiss albino mice

Test extracts	Dose (mg/kg/day)	Packed cell volume		% Change
		Before treatment (D_0_)	After treatment (D_4_)	
Aqueous	NC	51.13 ± 0.62	40.35 ± 0.26*	− 21.08^a^
	200	50.83 ± 0.79	50.31 ± 0.93	− 1.02^b^
	400	51.42 ± 0.64	50.68 ± 0.63	− 1.44^b^
	600	52.30 ± 0.27	51.93 ± 0.36	− 0.71^b^
	PC	53.06 ± 0.60	52.84 ± 0.52	− 0.41^b^
Methanol	NC	53.2 ± 0.31	43.28 ± 0.85*	− 18.65^a^
	200	51.68 ± 0.45	50.81 ± 0.46	− 1.68^b^
	400	51.03 ± 0.09	50.29 ± 0.49	− 1.45^b^
	600	52.05 ± 0.36	51.38 ± 0.27	− 1.29^b^
	PC	50.78 ± 0.39	51.05 ± 0.08	0.53^b^
Chloroform	NC	50.92 ± 0.55	44.01 ± 1.53*	− 13.57^a^
	200	51.67 ± 0.25	51.02 ± 0.46	− 1.26^b^
	400	52.3 ± 0.36	52.12 ± 0.33	− 0.34^b^
	600	53.16 ± 0.63	53.13 ± 0.64	− 0.06^b^
	PC	51.42 ± 0.35	52.08 ± 0.55	1.28^b^

Values are presented as Mean ± SEM

PC, positive control; NC, negative control (0.2 ml of 3% Tween 80)

^a,b^Values in the same column followed by the same letter do not differ significantly (P > 0.05)

Values shown by * are significant at  P < 0.05

### Mean survival time

All the experimental mice treated with the different extracts had increased dose dependent mean survival days (e.g. the chloroform extract exhibited the maximum MST of 12.53 at 600 mg/kg b.wt) as compared to the respective negative control groups (the maximum MST was 7.93). But all the mice treated with CQ survived for 3 months (Additional file 1).

### Discussion

All the test leaf extracts of aqueous, methanol and chloroform of *V. adoensis* have shown different degrees of parasitaemia inhibition in dose-related fashion. The highest suppression was recorded in the methanol extract treated mice (83.36% at the oral dose of 600 mg/kg). Previous studies have demonstrated the presence of secondary metabolites such as alkaloids, steroids, saponins, flavonoides, anthraquinones, terpenoids, sterols, diterpenoid, glycosides, tannins and sesquiterpene lactones in* Vernonia* species [7, 19]. The parasitaemia inhibition could be attributed to the presence of these metabolites.

Furthermore, the present result is in agreement with the result of previous in vivo study by Abosi and Raseroka [20] that reported suppressive effect of ethanolic extracts from the leaves and root bark of *Vernonia amygdalina*, where the leaf extract at 500 mg/kg resulted in 67% suppression of parasitaemia while the root-bark extract exerted 53.5% suppression at the same dose.

Similarly, a study carried out by Melariri and co-workers [21] showed a marked growth inhibition of parasites with values of 85% and 95% by the combination of dichloromethane extracts of leaves of *Cymbopogon citrates* and *V. amygdalina* at dose levels of 400 and 600 mg/kg b.wt against chloroquine sensitive strains of *P. berghei* in mice respectively. *V. amygdalina* leaves dichloromethane extract alone exerted 95.8% parasitaemia suppression at a higher dose of 800 mg/kg. In addition, the aqueous crude extract of the aerial part of *V. ambigua* **had** significant (P < 0.05) parasitaemia inhibition in a dose dependent suppression of parasite growth [10].

The statistical multiple comparison of the effect of each extracts on the body weight and PCV among groups on the 5th day of post-treatment and between D_0_ and D_4_ have shown that the two parameters to be within the normal range of values established for mice by Flecknell [15], adult body weight of 25–40 g and PCV of 32–54%. Therefore, the absence of any significant differences in the body weight and PCV parameters provides a support for the safety (non-toxic) of *V. adoensis* at all doses administered to the experimental mice. Previous studies on different species of Vernonia that also includes the present work have justified the potential of this genus as an antiplasmodial agent.

In conclusion, the crude leaves extract of *V. adoensis* have demonstrated antiplasmodial effect in Swiss albino mice. *P. berghei* infection is suppressed in a dose-dependent manner. The traditional use of the plant has thus some relevance based on this study.

## Limitations

The study was done only on the leaves of the plant and also only on crude extracts. It will be good if sub-fractions of the different extracts are tried. In addition using other parts of the plant such as the roots and flowers would have been more useful to come up with strong conclusion.

## Additional file

**Additional file 1.** Kaplan–Meier analysis.

## Abbreviations

AAU: Addis Ababa University
ANOVA: analysis of variance
b.wt: body weight
CQ: chloroquine
D_0_: day zero
D_4_: day four
MST: mean survival time
M ± SEM: mean plus/minus standard error of the mean
NC: negative control
PCV: packed cell volume
PC: positive control
RBC: red blood cells

## References

[1] Ayele DG, Zewotir TT, Mwambi HG (2012). Prevalence and risk factors of malaria in Ethiopia. Malaria J..

[2] RBM progress and impact series. World Malaria Day: Africa Update, Roll Back Malaria. 2010.

[3] Schlagenhauf P, Petersen E (2008). Malaria chemoprophylaxis: strategies for risk groups. Clin Microbiol Rev.

[4] WHO: Key facts about malaria. Geneva: World Health Organization; 2018.

[5] Geleta G, Ketema T. Severe Malaria Associated with *Plasmodium falciparum* and *P. vivax* among Children in Pawe Hospital, Northwest Ethiopia. Malaria Research and Treatment, Hindawi Publishing Corporation. 2016. p. 1–7. 10.1155/2016/1240962 PMC4800101 27047701

[6] Achan OA, Talisuna A, Erhart A, Yeka A, Tibenderana K, Baliraine N, Rosenthal J, Alessandro D (2011). Quinine, an old anti-malarial drug in a modern world: role in the treatment of malaria. Malaria J..

[7] Builders IM, Wannang NN, Ajoku AG, Builders FP, Orisadipe A, Aguiyi CJ (2011). Evaluation of the antimalarial potential of *Vernonia ambigua* Kotschy and Peyr (Asteraceae). Int J Pharmacol.

[8] Whegang Y, Tahar R, Foumane N, Soula G, Gwét H, Thalabard C, Basco K (2010). Efficacy of non-artemisinin and artemisinin-based combination therapies for uncomplicated falciparum malaria in Cameroon. Malaria J..

[9] Chitemere A, Mukanganyama S (2011). In vitro antibacterial activity of selected medicinal plants from Zimbabwe. Afr J Plant Sci Biotechnol.

[10] Dondorp AM, Nosten F, Yi P, Das D, Phyo AP, Tarning J (2009). Artemisinin resistance in *Plasmodium falciparum* malaria. N Engl J Med..

[11] Amato R, Lim P, Miotto O, Amaratunga C, Dek D (2017). Genetic markers associated with dihydroartemisinin-piperaquine failure in *Plasmodium falciparum* malaria in Cambodia: a genotype-phenotype association study. Lancet Infect Dis.

[12] Dalziel JM. The useful plants of West Tropical Africa. Being an appendix to the flora of West Tropical Africa. Crown Agents for the Colonies, London. 1955. p. 612.

[13] Fowler G. Traditional fever remedies. A list of zambian plants. 2006.

[14] Opoku R, Nethengwe F, Dludla P, Madida T, Shonhai A, Smith P, Singh M (2011). Larvicidal, antipyretic and antiplasmodial activity of some zulu medicinal plants. Planta Med.

[15] Flecknell PA, Tuffery A (1987). Non-surgical experimental procedures. Laboratory animals: an introduction for new experimenters.

[16] Tekalign D, Mekonnen Y, Animut A (2010). *In Vivo* Antimalarial Activities of *Clerodendrum myricoides*, *Dodonea angustifolia* and *Aloe debrana* Against *Plasmodium berghei*. Ethiop J Health Dev..

[17] Zerihun TM, Petros B, Mekonnen Y (2013). Evaluation of anti-plasmodial activity of crude and column fractions of extracts from *Withania somnifera*. Turk J Biol.

[18] Peter W, Portus H, Robinson L (1975). The four-day suppressive in vivo antimalarial test. Ann Trop Med Parasitol.

[19] Ajayia E, Adelekeb T, Adewumia M, Adeyemia A (2017). Antiplasmodial activities of ethanol extracts of *Euphorbia hirta* whole plant and *Vernonia amygdalina* leaves in *Plasmodium berghei*-infected mice. J Taibah Univ Sci..

[20] Abosi AO, Raseroka BH (2003). *In vivo* antimalarial activity of *Vernonia amygdalina*. Br J Biomed Sci.

[21] Melariri P, Campbell W, Etusim P, Smith P (2011). In vitro and in vivo antiplasmodial activities of extracts of *Cymbopogon citratus* staph and *Vernonia amygdalina* delile leaves. J Nat Prod.

