# Supplementary material for: Antiplasmodial activity of Vernonia adoensis aqueous, methanol and chloroform leaf extracts against chloroquine sensitive strain of Plasmodium berghei in vivo in mice
Source: BMC Res Notes. 2018 Oct 17;11:736. doi: 10.1186/s13104-018-3835-2 (PMC6192321; doi:10.1186/s13104-018-3835-2)

| **Case Processing Summary^a^** | | | | |
| --- | --- | --- | --- | --- |
| dose | Total N | N of Events | Censored | |
|  |  |  | N | Percent |
| 0 | 5 | 5 | 0 | 0.0% |
| 1 | 5 | 5 | 0 | 0.0% |
| 2 | 5 | 5 | 0 | 0.0% |
| 3 | 5 | 5 | 0 | 0.0% |
| Overall | 20 | 20 | 0 | 0.0% |
| a. treat = 1 | | | | |

| **Survival Table^a^** | | | | | | | |
| --- | --- | --- | --- | --- | --- | --- | --- |
| dose | | Time | Status | Cumulative Proportion Surviving at the Time | | N of Cumulative Events | N of Remaining Cases |
|  |  |  |  | Estimate | Std. Error |  |  |
| 0 | 1 | 5.000 | 1 | .800 | .179 | 1 | 4 |
|  | 2 | 7.000 | 1 | .600 | .219 | 2 | 3 |
|  | 3 | 8.000 | 1 | .400 | .219 | 3 | 2 |
|  | 4 | 9.000 | 1 | . | . | 4 | 1 |
|  | 5 | 9.000 | 1 | .000 | .000 | 5 | 0 |
| 1 | 1 | 8.000 | 1 | .800 | .179 | 1 | 4 |
|  | 2 | 9.000 | 1 | . | . | 2 | 3 |
|  | 3 | 9.000 | 1 | .400 | .219 | 3 | 2 |
|  | 4 | 11.000 | 1 | .200 | .179 | 4 | 1 |
|  | 5 | 12.000 | 1 | .000 | .000 | 5 | 0 |
| 2 | 1 | 10.000 | 1 | .800 | .179 | 1 | 4 |
|  | 2 | 11.000 | 1 | .600 | .219 | 2 | 3 |
|  | 3 | 12.000 | 1 | . | . | 3 | 2 |
|  | 4 | 12.000 | 1 | .200 | .179 | 4 | 1 |
|  | 5 | 13.000 | 1 | .000 | .000 | 5 | 0 |
| 3 | 1 | 12.000 | 1 | .800 | .179 | 1 | 4 |
|  | 2 | 13.000 | 1 | .600 | .219 | 2 | 3 |
|  | 3 | 14.000 | 1 | .400 | .219 | 3 | 2 |
|  | 4 | 15.000 | 1 | .200 | .179 | 4 | 1 |
|  | 5 | 16.000 | 1 | .000 | .000 | 5 | 0 |
| a. treat = 1 | | | | | | | |

| **Means and Medians for Survival Time^a^** | | | | | | | | |
| --- | --- | --- | --- | --- | --- | --- | --- | --- |
| dose | Mean^b^ | | | | Median | | | |
|  | Estimate | Std. Error | 95% Confidence Interval | | Estimate | Std. Error | 95% Confidence Interval | |
|  |  |  | Lower Bound | Upper Bound |  |  | Lower Bound | Upper Bound |
| 0 | 7.600 | .748 | 6.133 | 9.067 | 8.000 | 1.095 | 5.853 | 10.147 |
| 1 | 9.800 | .735 | 8.360 | 11.240 | 9.000 | .548 | 7.926 | 10.074 |
| 2 | 11.600 | .510 | 10.601 | 12.599 | 12.000 | .447 | 11.123 | 12.877 |
| 3 | 14.000 | .707 | 12.614 | 15.386 | 14.000 | 1.095 | 11.853 | 16.147 |
| Overall | 10.750 | .624 | 9.528 | 11.972 | 11.000 | 1.112 | 8.820 | 13.180 |
| a. treat = 1 | | | | | | | | |
| b. Estimation is limited to the largest survival time if it is censored. | | | | | | | | |

| **Overall Comparisons^a^** | | | |
| --- | --- | --- | --- |
|  | Chi-Square | df | Sig. |
| Log Rank (Mantel-Cox) | 22.122 | 3 | .000 |
| Breslow (Generalized Wilcoxon) | 19.771 | 3 | .000 |
| Tarone-Ware | 20.944 | 3 | .000 |
| Test of equality of survival distributions for the different levels of dose. | | | |
| a. treat = 1 | | | |


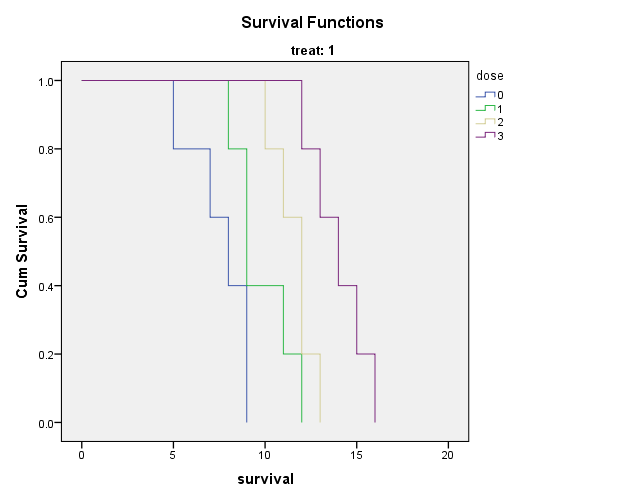


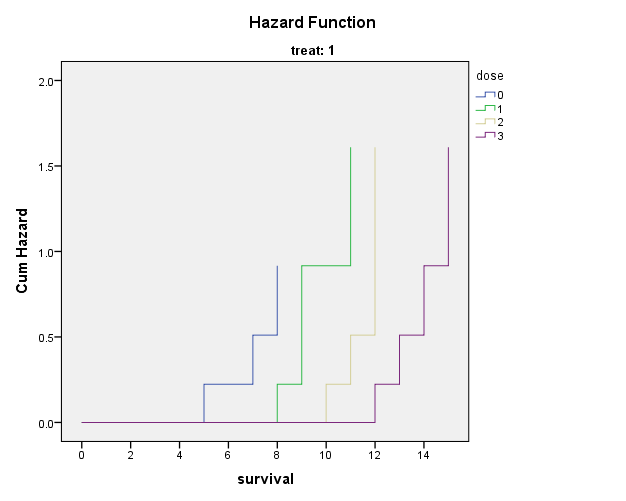


**treat = 2**

| **Case Processing Summary^a^** | | | | |
| --- | --- | --- | --- | --- |
| dose | Total N | N of Events | Censored | |
|  |  |  | N | Percent |
| 0 | 5 | 5 | 0 | 0.0% |
| 1 | 5 | 5 | 0 | 0.0% |
| 2 | 5 | 5 | 0 | 0.0% |
| 3 | 5 | 5 | 0 | 0.0% |
| Overall | 20 | 20 | 0 | 0.0% |
| a. treat = 2 | | | | |

| **Survival Table^a^** | | | | | | | |
| --- | --- | --- | --- | --- | --- | --- | --- |
| dose | | Time | Status | Cumulative Proportion Surviving at the Time | | N of Cumulative Events | N of Remaining Cases |
|  |  |  |  | Estimate | Std. Error |  |  |
| 0 | 1 | 6.000 | 1 | .800 | .179 | 1 | 4 |
|  | 2 | 7.000 | 1 | .600 | .219 | 2 | 3 |
|  | 3 | 8.000 | 1 | . | . | 3 | 2 |
|  | 4 | 8.000 | 1 | .200 | .179 | 4 | 1 |
|  | 5 | 9.000 | 1 | .000 | .000 | 5 | 0 |
| 1 | 1 | 9.000 | 1 | . | . | 1 | 4 |
|  | 2 | 9.000 | 1 | .600 | .219 | 2 | 3 |
|  | 3 | 10.000 | 1 | .400 | .219 | 3 | 2 |
|  | 4 | 11.000 | 1 | .200 | .179 | 4 | 1 |
|  | 5 | 12.000 | 1 | .000 | .000 | 5 | 0 |
| 2 | 1 | 10.000 | 1 | .800 | .179 | 1 | 4 |
|  | 2 | 11.000 | 1 | .600 | .219 | 2 | 3 |
|  | 3 | 12.000 | 1 | . | . | 3 | 2 |
|  | 4 | 12.000 | 1 | .200 | .179 | 4 | 1 |
|  | 5 | 13.000 | 1 | .000 | .000 | 5 | 0 |
| 3 | 1 | 11.000 | 1 | .800 | .179 | 1 | 4 |
|  | 2 | 12.000 | 1 | .600 | .219 | 2 | 3 |
|  | 3 | 13.000 | 1 | .400 | .219 | 3 | 2 |
|  | 4 | 14.000 | 1 | .200 | .179 | 4 | 1 |
|  | 5 | 16.000 | 1 | .000 | .000 | 5 | 0 |
| a. treat = 2 | | | | | | | |

| **Means and Medians for Survival Time^a^** | | | | | | | | |
| --- | --- | --- | --- | --- | --- | --- | --- | --- |
| dose | Mean^b^ | | | | Median | | | |
|  | Estimate | Std. Error | 95% Confidence Interval | | Estimate | Std. Error | 95% Confidence Interval | |
|  |  |  | Lower Bound | Upper Bound |  |  | Lower Bound | Upper Bound |
| 0 | 7.600 | .510 | 6.601 | 8.599 | 8.000 | .447 | 7.123 | 8.877 |
| 1 | 10.200 | .583 | 9.057 | 11.343 | 10.000 | 1.095 | 7.853 | 12.147 |
| 2 | 11.600 | .510 | 10.601 | 12.599 | 12.000 | .447 | 11.123 | 12.877 |
| 3 | 13.200 | .860 | 11.514 | 14.886 | 13.000 | 1.095 | 10.853 | 15.147 |
| Overall | 10.650 | .554 | 9.565 | 11.735 | 11.000 | .730 | 9.569 | 12.431 |
| a. treat = 2 | | | | | | | | |
| b. Estimation is limited to the largest survival time if it is censored. | | | | | | | | |

| **Overall Comparisons^a^** | | | |
| --- | --- | --- | --- |
|  | Chi-Square | df | Sig. |
| Log Rank (Mantel-Cox) | 26.080 | 3 | .000 |
| Breslow (Generalized Wilcoxon) | 23.972 | 3 | .000 |
| Tarone-Ware | 25.014 | 3 | .000 |
| Test of equality of survival distributions for the different levels of dose. | | | |
| a. treat = 2 | | | |


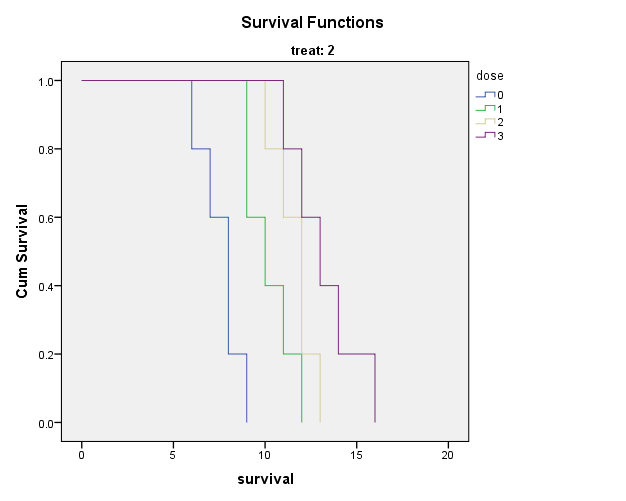


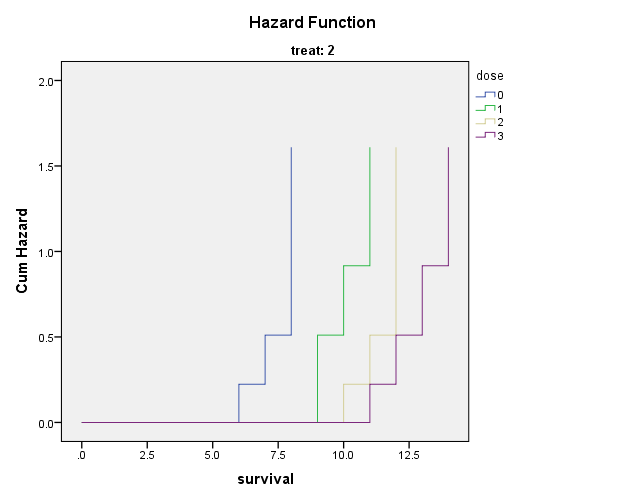


**treat = 3**

| **Case Processing Summary^a^** | | | | |
| --- | --- | --- | --- | --- |
| dose | Total N | N of Events | Censored | |
|  |  |  | N | Percent |
| 0 | 5 | 5 | 0 | 0.0% |
| 1 | 5 | 5 | 0 | 0.0% |
| 2 | 5 | 5 | 0 | 0.0% |
| 3 | 5 | 5 | 0 | 0.0% |
| Overall | 20 | 20 | 0 | 0.0% |
| a. treat = 3 | | | | |

| **Survival Table^a^** | | | | | | | |
| --- | --- | --- | --- | --- | --- | --- | --- |
| dose | | Time | Status | Cumulative Proportion Surviving at the Time | | N of Cumulative Events | N of Remaining Cases |
|  |  |  |  | Estimate | Std. Error |  |  |
| 0 | 1 | 6.000 | 1 | .800 | .179 | 1 | 4 |
|  | 2 | 7.000 | 1 | . | . | 2 | 3 |
|  | 3 | 7.000 | 1 | .400 | .219 | 3 | 2 |
|  | 4 | 8.000 | 1 | . | . | 4 | 1 |
|  | 5 | 8.000 | 1 | .000 | .000 | 5 | 0 |
| 1 | 1 | 8.000 | 1 | .800 | .179 | 1 | 4 |
|  | 2 | 9.000 | 1 | . | . | 2 | 3 |
|  | 3 | 9.000 | 1 | .400 | .219 | 3 | 2 |
|  | 4 | 11.000 | 1 | .200 | .179 | 4 | 1 |
|  | 5 | 12.000 | 1 | .000 | .000 | 5 | 0 |
| 2 | 1 | 9.000 | 1 | .800 | .179 | 1 | 4 |
|  | 2 | 10.000 | 1 | . | . | 2 | 3 |
|  | 3 | 10.000 | 1 | .400 | .219 | 3 | 2 |
|  | 4 | 11.000 | 1 | .200 | .179 | 4 | 1 |
|  | 5 | 12.000 | 1 | .000 | .000 | 5 | 0 |
| 3 | 1 | 11.000 | 1 | . | . | 1 | 4 |
|  | 2 | 11.000 | 1 | .600 | .219 | 2 | 3 |
|  | 3 | 12.000 | 1 | . | . | 3 | 2 |
|  | 4 | 12.000 | 1 | .200 | .179 | 4 | 1 |
|  | 5 | 13.000 | 1 | .000 | .000 | 5 | 0 |
| a. treat = 3 | | | | | | | |

| **Means and Medians for Survival Time^a^** | | | | | | | | |
| --- | --- | --- | --- | --- | --- | --- | --- | --- |
| dose | Mean^b^ | | | | Median | | | |
|  | Estimate | Std. Error | 95% Confidence Interval | | Estimate | Std. Error | 95% Confidence Interval | |
|  |  |  | Lower Bound | Upper Bound |  |  | Lower Bound | Upper Bound |
| 0 | 7.200 | .374 | 6.467 | 7.933 | 7.000 | .548 | 5.926 | 8.074 |
| 1 | 9.800 | .735 | 8.360 | 11.240 | 9.000 | .548 | 7.926 | 10.074 |
| 2 | 10.400 | .510 | 9.401 | 11.399 | 10.000 | .548 | 8.926 | 11.074 |
| 3 | 11.800 | .374 | 11.067 | 12.533 | 12.000 | .447 | 11.123 | 12.877 |
| Overall | 9.800 | .451 | 8.917 | 10.683 | 10.000 | 1.112 | 7.820 | 12.180 |
| a. treat = 3 | | | | | | | | |
| b. Estimation is limited to the largest survival time if it is censored. | | | | | | | | |

| **Overall Comparisons^a^** | | | |
| --- | --- | --- | --- |
|  | Chi-Square | df | Sig. |
| Log Rank (Mantel-Cox) | 23.489 | 3 | .000 |
| Breslow (Generalized Wilcoxon) | 22.311 | 3 | .000 |
| Tarone-Ware | 22.958 | 3 | .000 |
| Test of equality of survival distributions for the different levels of dose. | | | |
| a. treat = 3 | | | |


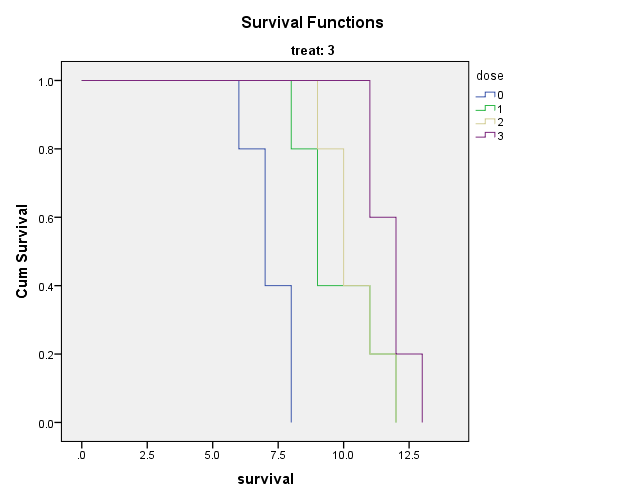


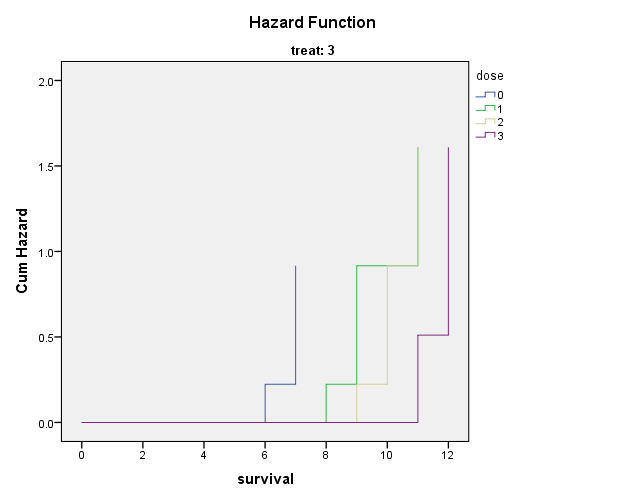

Supplement: Supplementary file 1 — Additional file 1. Kaplan–Meier analysis. [file 13104_2018_3835_MOESM1_ESM.doc]
